# Vascularized drusen: a cross-sectional study

**DOI:** 10.1186/s40942-019-0187-6

**Published:** 2019-08-20

**Authors:** Chris Or, Jeffrey S. Heier, David Boyer, David Brown, Sumit Shah, Agha Yasin Alibhai, James G. Fujimoto, Nadia Waheed

**Affiliations:** 1 New England Eye Center, Tufts Medical Center, 260 Tremont Street, Biewend Building, 9–11th Floor, Boston, MA 02111 USA; 2 Ophthalmic Consultants of Boston, Boston, MA USA; 3 Retina-Vitreous Associates Medical Group, Beverly Hills, CA USA; 4 Retina Consultants of Houston, Houston, TX USA; 5 NJ Retina, New Brunswick, NJ USA; 6 Department of Electrical Engineering and Computer Science, and Research Laboratory of Electronics, Massachusetts Institute of Technology, Cambridge, MA USA

**Keywords:** Age-related macular degeneration, Retina, Optical coherence tomography, Optical coherence tomography angiography, Vascularized drusen, Retinal imaging

## Abstract

**Background:**

To investigate whether neovascularization may arise and be detectable in drusen, as reported in histopathologic studies, by OCTA prior to developing exudation and to assess its prevalence in a cohort of patients with intermediate AMD.

**Methods:**

Retrospective cross-sectional study of 128 patients with intermediate AMD recruited as part of a separate ongoing clinical trial conducted at multiple large tertiary referral retina clinics. One hundred and twenty-eight consecutive patients with exudative AMD in one eye and intermediate non-exudative AMD in the fellow eye were enrolled and analyzed between September 2015 and March 2017.

**Results:**

SD-OCTA identified vascularization within drusen in 7 of 128 eyes, for a prevalence of 5.5%. A total of 12 instances of vascularized drusen were noted. Out of the 12 vascularized drusen noted, 7 were located in the parafoveal region or subfoveal region and 5 was in the extrafoveal region. 9 of 12 instances of vascularized drusen exhibited a uniform sub-RPE hyperreflectivity, whilst 3 of 12 exhibited more heterogenous reflectivity. In all 12 instances, FA images failed to identify the neovascular nature of vascularized drusen.

**Conclusions:**

Our results demonstrate the utility of SD-OCTA for the diagnosis of vascularized drusen in patients with intermediate non-exudative AMD. Longitudinal studies are needed to delineate the evolution and conversion risk of these lesions over time, which can be of substantial clinical relevance.

## Background

Age-related macular degeneration (AMD) is one of the leading causes of vision loss [1]. The early stage of the disease is characterized by the presence of drusen, which consist of extracellular debris with varying composition located between the basal lamina of the RPE and inner layer of Bruch’s membrane [2]. Large, soft and confluent drusen have been associated with an increased risk of progression to advanced AMD [3, 4]. Approximately 80% of cases of vision loss secondary to advanced AMD are due to exudative AMD [5]. Early detection of CNV and early management of exudative AMD have been shown to be associated with better visual acuity outcomes in patients who convert to clinically significant exudative AMD [6].

Historically, exudative AMD, presenting as leakage from immature vessels, is diagnosed using fluorescein angiography (FA). Indocyanine green angiography (ICGA) is deemed the gold standard when assessing for the presence of type 1 neovascularization, owing to its improved penetration below the RPE.

Studies using ICG videography on patients with non-exudative AMD demonstrated that subclinical neovascularization could occur even in this cohort of patients [7]. A study by Hanutsaha et al. reported that 8% of patients with exudative AMD in the fellow eye also had ICGA evidence of MNV (plaques or hot spots) in the clinically non-exudative study eye. On follow up, eyes with these ‘quiescent’ or ‘non-exudative’ MNV had an increased risk of converting to exudative AMD compared to eyes without angiographic evidence of MNV [7]. This is further supported by histopathological studies performed by Sarks and Green et al. in post-mortem eyes of patients with non-exudative intermediate AMD, which revealed that upwards of 20% had evidence of neovascularization [8, 9].

However, ICGA is seldom utilized to monitor asymptomatic patients with intermediate AMD due to its associated risks and invasive nature. The recent advent of optical coherence tomography angiography (OCTA) technology provides a rapid and non-invasive method by which clinicians and researchers can diagnose and monitor non-exudative neovascularization in eyes with dry AMD [10]. Roisman et al. and Querques et al. were amongst the first to demonstrate the ability of OCTA to detect non-exudative neovascularization in patients with phenotypic dry AMD. They investigated and characterized treatment-naïve quiescent CNV in intermediate AMD using multimodal imaging, and described such lesions as demonstrating ill-defined hyperfluorescence with no leakage on FA, hypercyanescence in a plaque configuration on ICGA, and a shallow, irregular pigment epithelial detachment. These elevations of the retinal pigment epithelium (RPE) exhibited moderate hyperreflectivity and a major axis in the horizontal plane on structural optical coherence tomography (OCT) [11, 12]. Furthermore, ICGA revealed 3 asymptomatic plaques out of 11 eyes in their study, which were also observed on OCTA [11]. A follow up study performed by de Oliveira Dias et al. using SS-OCTA reported that 14.4% of patients with intermediate and late non-exudative AMD, with exudative AMD in the fellow eye, had evidence of subclinical macular neovascularization (MNV). Patients with phenotypic intermediate dry AMD, but who had subclinical MNVs carried an increased risk of conversion to exudative AMD compared to patients who did not have non-exudative MNV (21.1% vs. 5.4% at 1 year of follow up) [13].

Querques et al. reported long term follow up of a patient with a low-lying pigment epithelial detachment and subclinical neovascularization on OCTA [14]. On retrospective review of the images of this patient, there was a large dome-shaped drusen that then developed a low-lying pigment epithelial detachment and the patient was noted to have an MNV [14]. However, it is still not clear at which point the MNVs arise in the evolution of drusen and pigment epithelial detachments. In this study of patients with intermediate non-exudative AMD, we look to identify the earliest signs of non-exudative MNV and to investigate whether neovascularization may arise and be detectable in drusen as reported in histopathologic studies.

## Methods

This is a retrospective cross-sectional study conducted including patients with a diagnosis of non-exudative AMD in the study eye and exudative AMD in the fellow eye that were previously prospectively enrolled as part of an ongoing registered clinical trial (Clinicaltrials.gov identifier: NCT02462889). Inclusion criteria included: subjects with intermediate non-exudative AMD in the study eye marked by the presence of ten or more intermediate sized drusen (≥ 63 and < 125 μm), 1 or more large drusen (≥ 125 μm), and/or retinal pigmentary changes, and exudative AMD in the fellow eye. Exclusion criteria included a history of treatment for exudative AMD in the study eye or a history of serious ocular conditions or ophthalmic surgery. Patients received a complete ophthalmologic examination and multimodal imaging including color fundus photos, fluorescein angiography, OCT, and OCTA. OCTA imaging was performed using a spectral domain instrument, the AngioVue RTVue Avanti (software vers. 2017.1.0.151, Optovue, Fremont, California, USA), with both the 3 × 3 mm and 6 × 6 mm scan patterns centered on the fovea. All ophthalmic imaging was analyzed by 2 independent expert readers at the Boston Image Reading Center in a masked fashion, and the inter-rater reliability was assessed using Cohen’s Kappa. The presence of drusen and of intermediate AMD were noted on color fundus photographs. Fluorescein angiography images were analyzed for the presence of MNV. Drusen were identified on OCT and were included in the analysis. A cut off of a maximum diameter of 250 microns was used, so as to not inadvertently include low-lying pigment epithelial detachments. The OCT images were also carefully analyzed to exclude eyes with shallow, low-lying pigment epithelial detachments that may be associated with sub-clinical non-exudative MNV. OCTA images were then analyzed to confirm the presence or absence of neovascularization within the drusen. This was done by examining the individual B-scans with flow overlay and identifying flow in the individual druse. Care was taken to ensure that projection artifacts from superficial vessels were not mistaken for flow. The en face OCTA images were also analyzed to detect evidence of flow.

## Results

One hundred and twenty-eight consecutive patients with exudative AMD in one eye and intermediate non-exudative AMD in the fellow eye were enrolled and analyzed between September 2015 and March 2017.

### Prevalence and characteristics

**Fig. 1:**
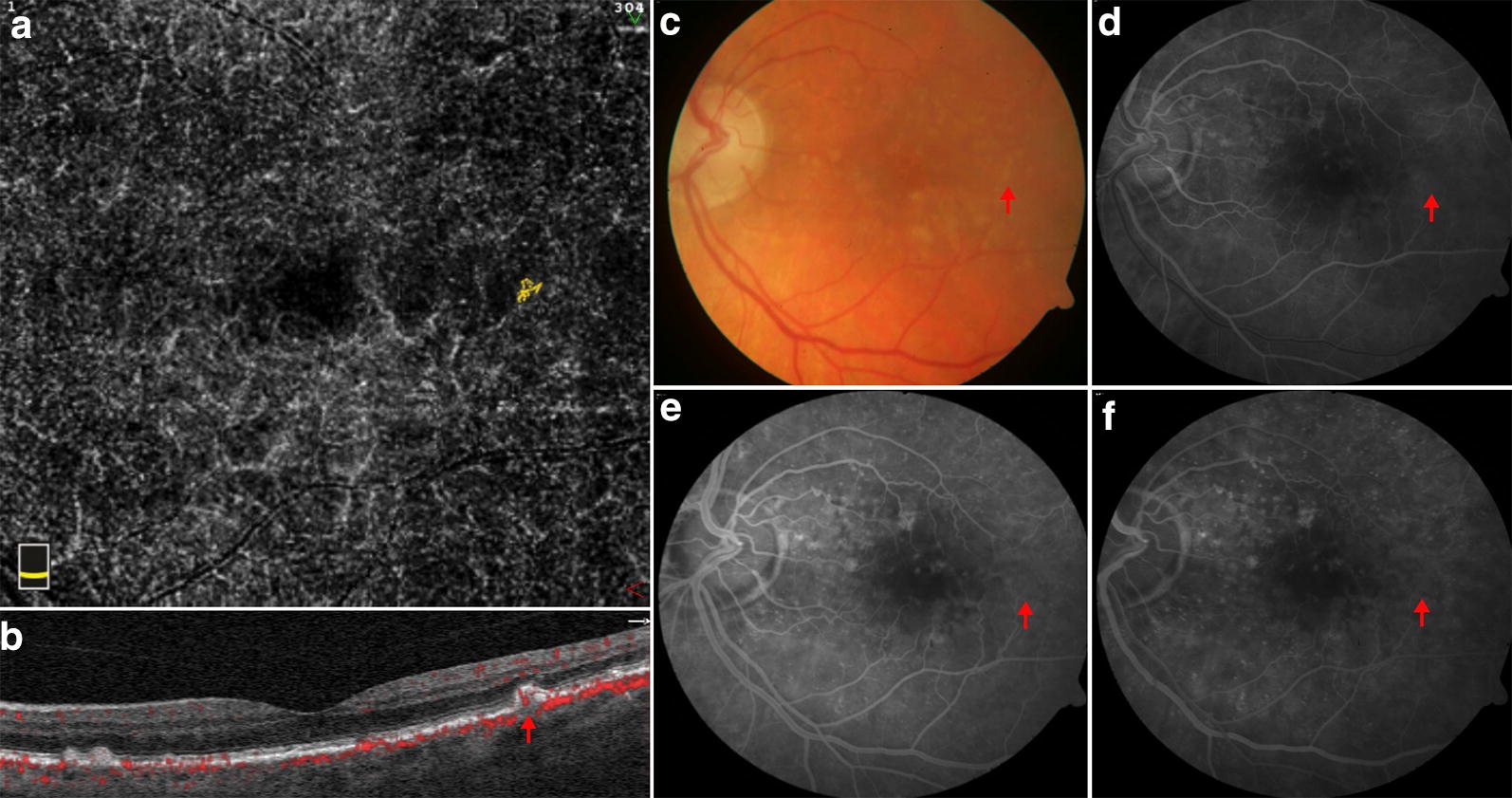
Vascularized druse on multimodal imaging. A typical yellow drusenoid lesion is noted on the color fundus image (**c** red arrow). On FA, there is minimal staining (**d**–**f** red arrow). Optical coherence tomography demonstrates a dome-shaped drusenoid retinal pigment epithelium (RPE) elevation with homogenous sub-RPE hyperreflectivity. Optical coherence tomography angiography en face image (**a**) shows a neovascular network, which corresponds with the flow signal (**b**) located within the drusenoid lesion. Manual segmentation of the RPE and Bruch’s membrane was used to clearly visualize the neovascular network

**Fig. 2:**
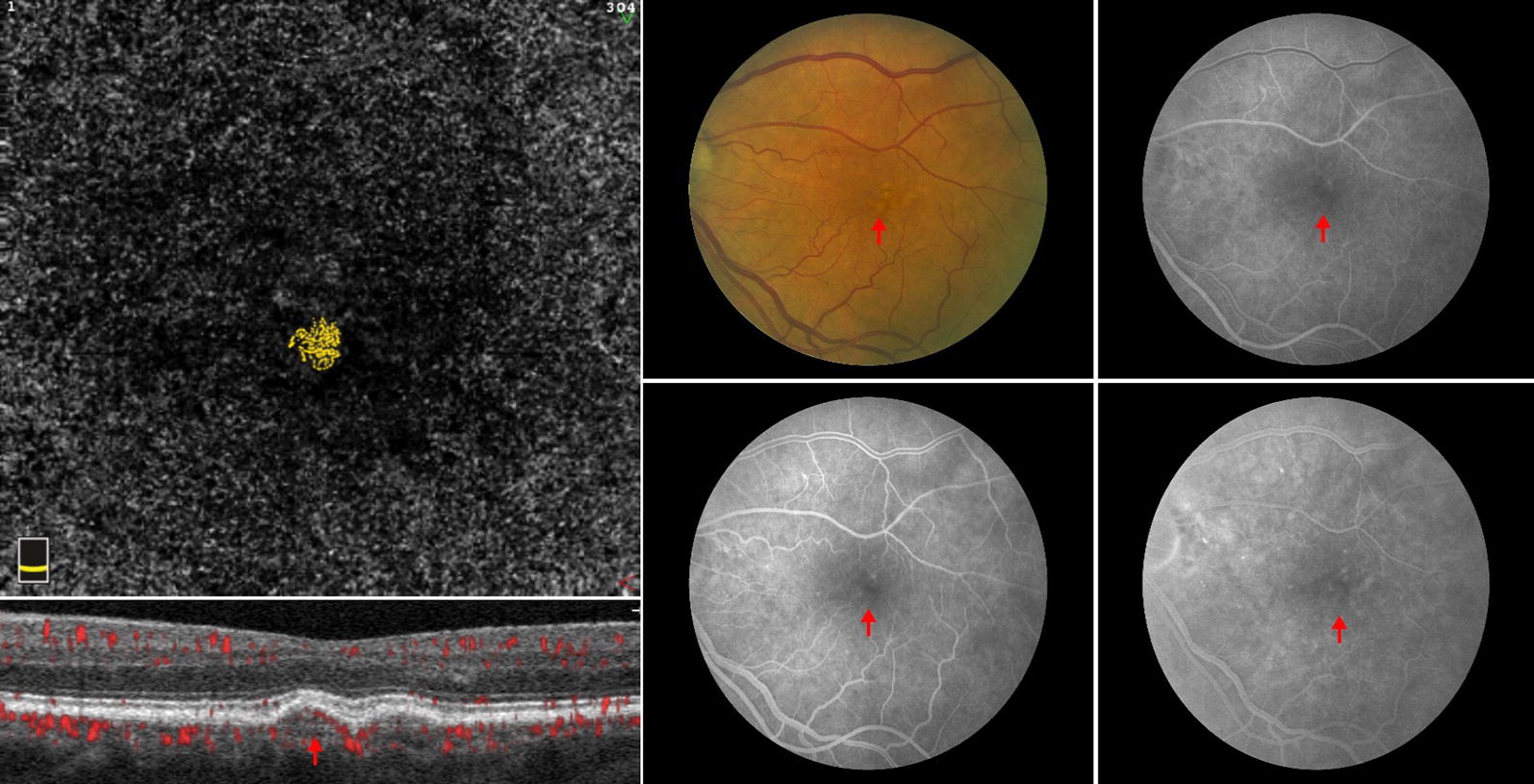
Vascularized druse on multimodal imaging. A typical yellow drusenoid lesion is noted on the color fundus image (**c** red arrow). On FA, there is minimal staining (**d**–**f** red arrow). Optical coherence tomography demonstrates a dome-shaped drusenoid retinal pigment epithelium (RPE) elevation with a heterogenous multi-laminar sub-RPE hyperreflectivity. Optical coherence tomography angiography en face image (**a**) shows a neovascular network, which corresponds with the flow signal (**b**) located within the drusenoid lesion. Manual segmentation of the RPE and Bruch’s membrane was used to clearly visualize the neovascular network

SD-OCTA identified vascularization within drusen in 7 of 128 eyes for a prevalence of 5.5%. A total of 12 instances of vascularized drusen were noted (Figs. 1, 2). The vascularization on these drusen was best visualized by evaluating serial B-scans with flow overlay. On the en face OCTA images, a clear neovascular network could not be visualized in most lesions using the default segmentation on the native software. Manual segmentation of the drusenoid RPE elevation provided an improved visualization of the neovascular network. A large variation in OCTA flow signal intensity was noted, with some exhibiting obvious flow signal within the drusenoid deposit (Fig. 2) and others presenting with more subtle findings (Fig. 1). Out of the 12 vascularized drusen noted, 7 were located in the parafoveal region or subfoveal region and 5 were in the extrafoveal region.

**Fig. 3:**
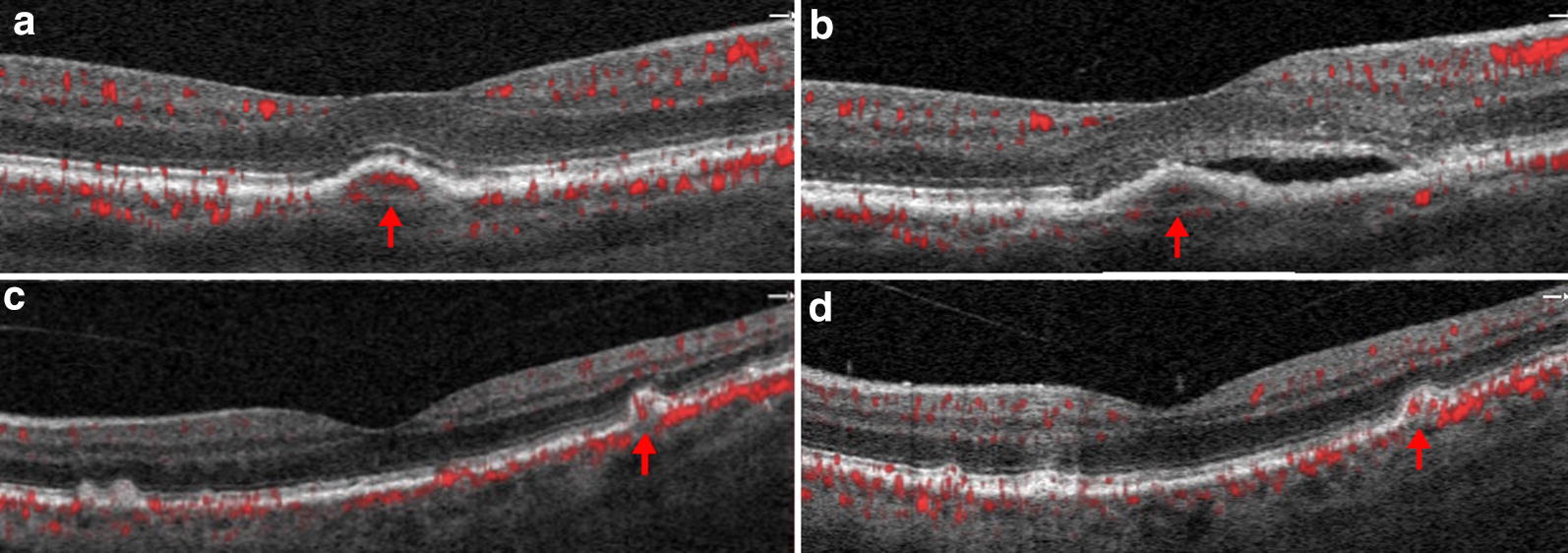
Vascularized druse on follow up. Vascularized druse identified at baseline (**a**) with development of a low-lying pigment epithelial detachment and subretinal fluid at 6 months follow up (**b**). Another instance of vascularized druse identified at baseline (**c**) with persisting vascularity within the lesion at 2 years follow up (**d**)

Nine of 12 instances of vascularized drusen exhibited a uniform sub-RPE hyperreflectivity (Fig. 2) whilst 3 of 12 exhibited more heterogenous reflectivity (Fig. 1). FA images of vascularized drusen demonstrated staining, but no leakage (Figs. 1, 2). In all 12 instances, FA images failed to identify the neovascular nature of vascularized drusen. Longitudinal follow up is available on two of our patients and demonstrates conversion of one patient to exudative AMD at 6 months follow up and stability of the other patient, with persistent vascularity observed within the lesion at 2 years follow up (Fig. 3).

The Cohen’s Kappa coefficient for the grading of ophthalmic images ranged from 0.80 to 0.85, indicating excellent inter-rater reliability.

## Discussion

To our knowledge this is the first reported case series on vascularized drusen. Herein, we analyzed 128 patients with intermediate non-exudative AMD with exudative AMD in the fellow eye and report a prevalence rate of 5.5% of vascularized drusen. Recent studies by Roisman et al., de Oliviera Dias et al., and Querques et al. have demonstrated that phenotypic dry AMD consists of two different OCT angiographic subtypes, a high-risk type with non-exudative subclinical MNV and a truly ‘dry’ type with no MNV. However, it is not clear at what stage the non-exudative MNV develops [11–13]. This study shows that neovascularization may be present as early as in drusen.

Interestingly, the majority of lesions included in our study displayed a uniform sub-RPE hyperreflectivity (9/12), with only 3/12 displaying a multi-laminar sub-RPE hyperreflectivity. It has been previously postulated that the multi-laminar sub-RPE hyperreflectivity observed in drusen may represent layers of lipid mineralization or a type 1 neovascularization [15, 16]. The identification of neovascularization in drusenoid lesions presenting with uniform sub-RPE hyperreflectivity suggests that OCT alone is insufficient for its detection. Furthermore, vascularized drusen could not be detected using FA or CFP. Querques et al. suggested that these lesions may be visible on ICG [14]. It remains a limitation of our study that ICGA results were not available to confirm our findings. However, given that ICGA is seldom used clinically, our results suggest that OCTA may be the modality of choice for detecting vascularized drusen, a notion supported previously in the literature [11, 13, 14].

**Fig. 4:**
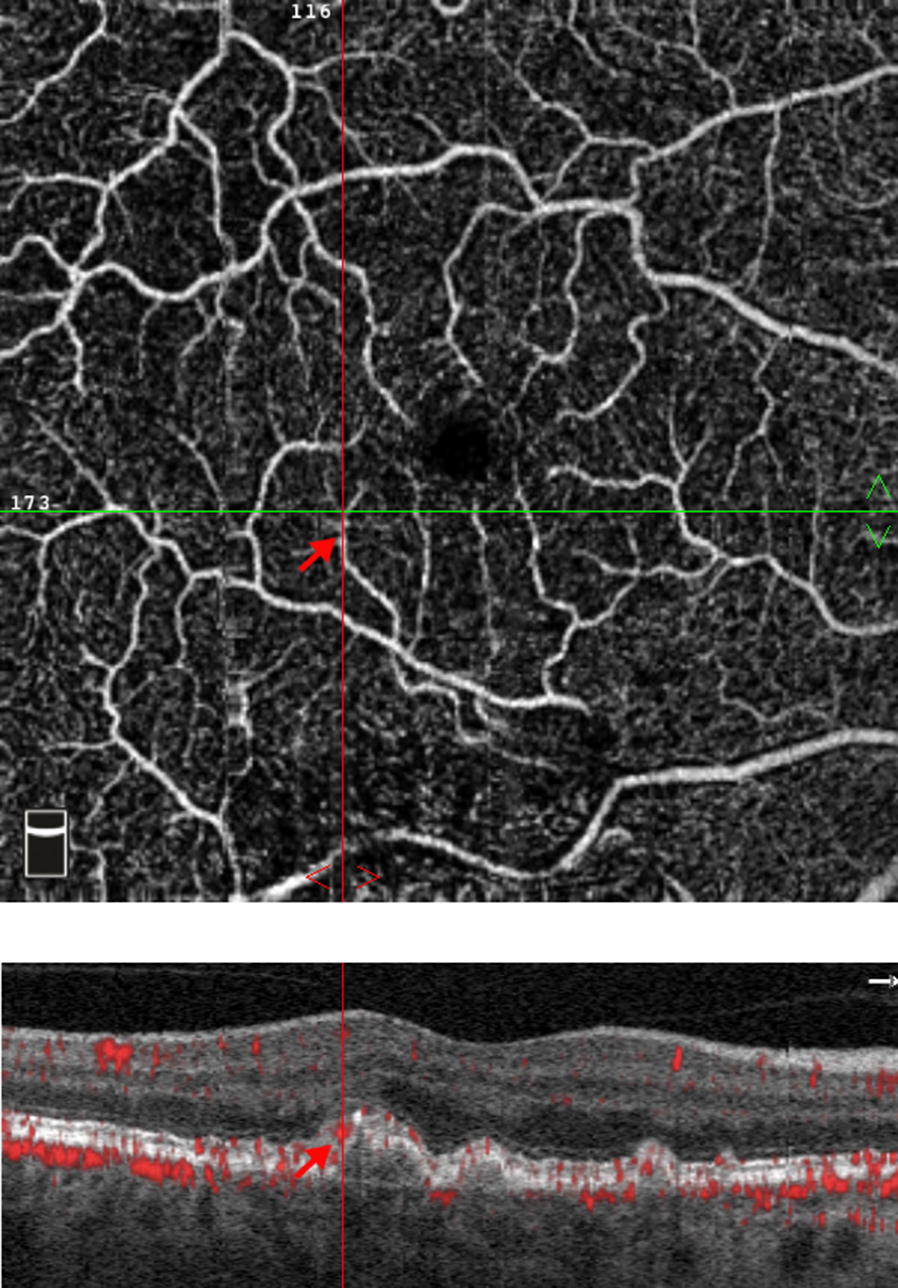
Example of artifact when identifying vascularized drusen. An en face optical coherence tomography angiography image of the superficial plexus is shown (top). The corresponding optical coherence tomography image of the cross section is shown with flow overlay (bottom). The red arrow in the bottom image demonstrates an example of projection artifact, originating from the blood vessel in the superficial image (red arrow; top image)

The interpretation of SD-OCTA scans, however, remains challenging due to the presence of artifacts and can be viewed as a limitation of our study. In particular, projection artifacts from superficial vessels can often mimic flow signals under the RPE and make it difficult to isolate and identify vascularized drusen [17]. Examination of sequential OCTA B-scans as well as en face OCTA images at different depths can help confirm findings. In our study, we used very stringent criteria to avoid misdiagnosing projection artifact as neovascularization and diagnosed as negative any cases that were questionable (Fig. 4). Therefore, the actual prevalence of vascularized drusen may be higher than has been noted in our study.

It is still not clear, based on this cross-sectional study, what the evolution of these vascularized drusen is, and whether they may regress or progress into and be a precursor for shallow, low-lying PEDs harboring non-exudative MNVs noted by previous studies, or whether vascularized drusen are an independent phenotype conferring a higher risk of conversion to exudative AMD. Longitudinal studies are needed to delineate the evolution of these lesions over time and can be of substantial clinical relevance.

## Conclusion

In summary, we report that 5.5% of 128 high risk intermediate AMD patients in this study have vascularized drusen. Most instances of vascularized drusen in our cohort had a uniform sub-RPE hyperreflectivity and a majority of the lesions were in the subfoveal and parafoveal regions. In all instances, FA failed to identify the presence of the neovascularization. Our results demonstrate the utility of SD-OCTA for the detection of vascularized drusen in patients with intermediate non-exudative AMD.

## Data Availability

The datasets used and/or analysed during the current study are available from the corresponding author on reasonable request.
